# Diet-dependent entropic assessment of athletes’ lifespan

**DOI:** 10.1017/jns.2021.78

**Published:** 2021-09-29

**Authors:** Cennet Yildiz, Melek Ece Öngel, Bayram Yilmaz, Mustafa Özilgen

**Affiliations:** 1 Department of Food Engineering, Yeditepe University, Kayısdagi, Atasehir, Istanbul 34755, Turkey; 2 Nutrition and Dietetics Department, Yeditepe University, Kayısdagi, Atasehir, Istanbul 34755, Turkey; 3 Faculty of Medicine, Department of Physiology, Yeditepe University, Istanbul, Turkey

**Keywords:** Athletes’ diet, Athletes’ longevity, Entropic age, Lifespan entropy

## Abstract

Life expectancies of the athletes depend on the sports they are doing. The entropic age concept, which was found successful in the previous nutrition studies, will be employed to assess the relation between the athletes’ longevity and nutrition. Depending on their caloric needs, diets are designed for each group of athletes based on the most recent guidelines while they are pursuing their careers and for the post-retirement period, and then the metabolic entropy generation was worked out for each group. Their expected lifespans, based on attaining the lifespan entropy limit, were calculated. Thermodynamic assessment appeared to be in agreement with the observations. There may be a significant improvement in the athletes’ longevity if they shift to a retirement diet after the age of 50. The expected average longevity for male athletes was 56 years for cyclists, 66 years for weightlifters, 75 years for rugby players and 92 years for golfers. If they should start consuming the retirement diet after 50 years of age, the longevity of the cyclists may increase for 7 years, and those of weightlifters, rugby players and golfers may increase for 22, 30 and 8 years, respectively.

## Introduction

During recent years, several articles have proposed the use of the entropy perspective to assess the physiological state of the human body under different physical conditions^(1–5)^. A fraction of the generated entropy when it accumulates in the system causes structural changes, as expressed by the ‘entropy theory of ageing’^(6)^. The results of these new studies assumed a new role in the term ‘entropy’. The ‘entropic age’ concept suggests that ageing-related changes in the body, such as loss of cellular functions and overwhelming of maintenance systems, may be explained in terms of entropy generation^(7–12)^. Physiological processes such as muscle contraction, nervous, gastrointestinal and reproductive functions, absorption of ions and detoxification of exogenous substances require consumption of ATP, and most of these processes are accompanied by dissipation of heat^(13–16)^. Silva and Annamalai^(6,17)^ made a significant contribution to this area of research by quantifying the entropy generation during ageing and related it to the ageing stress on individual organs^(18)^. Kuddusi^(19)^, after studying nutritional habits in seven climate zones of Turkey, employed the lifespan entropy concept to estimate the expected lifespan as a function of the local foods consumed by the inhabitants. Öngel *et al.*^(20)^ took this analysis one step further to find the thermodynamic bases for why women live longer than men do, after performing telomere-length-regulated and diet-based entropic assessment. Their estimation of the life expectancy of the women on four different diets showed that women had a longer lifespan than men in every diet. Faster shortening of the telomer lengths in men was the major reason for the shorter life expectancy. The highest and the lowest life expectancy for women were estimated with the Mediterranean and the vegetarian diets, respectively; men were estimated to have the longest lifespan with the vegetarian diet and the shortest lifespan with the ketogenic diet.

### Effects of the exercise types and entropy generation on athlete longevity

The longevity of an athlete is determined by the diet he/she is consuming, and the intensity of the dynamic or static exercise he/she is performing^(21–26)^. In dynamic exercise, moderate muscle length changes and moderate joint movement through rhythmic contractions cause moderately small intramuscular force changes. Static exercise does not involve muscle length alterations or joint movements, and it is based on the expansion of a moderately small intramuscular force with little change of muscle length. Classification of sports based on the fraction of their static and dynamic components is shown in Table 1. The increasing dynamic component is defined in terms of the estimated percent of maximal oxygen uptake achieved, which results in an increased cardiac output. The increasing static component is related to the estimated percent of maximal voluntary contraction of the muscles reached, which results in increased blood pressure^(28,29)^.

**Table 1.** Classification of the sports based on their static and dynamic components

	Low-dynamic component (<40 % Max O_2_)	Moderate dynamic component (40–70 % Max O_2_)	High-dynamic component (>70 % Max O_2_)
High-static component (>50 % MVC)	Gymnastics, sailing windsurfing, **weightlifting**	Skateboarding, wrestling, snowboarding, bodybuilding	Boxing, canoeing/kayaking, **cycling,** rowing
Moderate static component (20–50 % MVC)	Archery, auto-racing, diving, motorcycling	**Rugby,** figure skating, surfing, running (sprint)	Basketball, swimming, running (middle distance), handball
Low-static component (<20 % MVC)	Billards, bowling, **golfing,** curling	Baseball/softball, table tennis, volleyball, fencing	Badminton, race walking, soccer, tennis

Summarised from Mitchell and Wildenthal^(27)^.

Dynamic exercise causes an increase in oxygen demand, arterial pressure and cardiac output due to high metabolic demand in the contracting muscle. Likewise, blood pressure and oxygen uptake increase with static exercise^(27)^. The high jumpers and marathon runners usually have low body weights and tend to live longer than the general population; on the other hand, 100 m sprinters are believed to live less than the general population. Within this context, powerlifters are seen to have the shortest life expectancy mainly because of their high body weight^(30)^.

All sports, depending on muscular activity, increase the energy expenditure rate. Therefore, nutrient requirement raises to supply the energy demand of the body. Depending on the metabolism of macronutrients such as carbohydrates, amino acids and fats in the presence of O_2_, the dissipation of the heat that is released as a result of doing sports increases. This process leads to metabolic entropy generation. All living organisms maintain their lives far from thermal, chemical and mechanical equilibrium. They attain equilibrium with the environment only at death. Organisms avoid entropy accumulation to sustain themselves. The entropy generation rate is related to ageing and enables the prediction of lifespan^(6,17,25,31–35)^.

### Nutrition recommendations for athletes

Nutrition has a very significant share in the efforts for maximising the effect of training of the athletes. A moderately active person may easily acquire his/her recommended daily nutrition, whereas for athletes, ‘what’, ‘when’ and ‘how much’ they eat matters^(36)^. Athletes, who perform the moderately intense exercise for 2–3 h a day and 5–6 times a week and high-intensity exercise for 3–6 h/d and 5–6 d a week, may spend about 1200 kcal/h of exercise. On the other hand, the calorie need of elite athletes may increase, for instance, the energy expenditure of a cyclist may be 8500–10 000 kcal on a competition day^(36)^. Failing to meet the nutritional recommendations may lead to unwanted weight loss which may be a problem, especially with cyclists, swimmers, boxers, dancers, etc.^(37)^ To sustain the muscle mass and increased performance, athletes must uptake sufficient macronutrients, carbohydrates, proteins and fats. If an athlete is performing moderate exercise, it is optimal to consume 55–60 % of the daily calories as carbohydrates, which may correspond to 5–8 g/kg per d. Athletes are recommended to consume 1⋅5–2 g/kg per d of proteins to meet the required amount^(37)^. Dietary fat consumption recommended for the athletes to maintain and promote their health is the same as that of the general population^(37)^, which is 25–30 % of the daily calorie intake^(38)^.

Timing of the nutrient uptake is of prime concern for optimal nutrition of the athletes. Muscle glycogen levels and available carbohydrates in the body are major prevailing factors for exercise performance for pace, duration and work output. It is stated that, if the exercise should be longer than 60 min, carbohydrates intake would be important to maintain the blood sugar and muscle glycogen levels, and should be about 30–60 g/h, which can be provided as 6–8 % carbohydrate solution. Different combinations of carbohydrate sources can be included in the pre-exercise meal, but high amounts of fructose are not recommended to avoid gastrointestinal problems. Whereas post-exercise meals, combining carbohydrates and proteins, are recommended to reduce muscle loss and have a beneficial effect on the recovery of muscle glycogen levels^(39)^.

Athletes would not require any additional vitamin and mineral supplementation if they consume the right variety and amounts of fruits and vegetables. Their fluid intake should be monitored since a fluid loss would cause electrolyte imbalance. Athletes must meet their fluid requirements to improve their exercise performance, muscle healing and blood sugar balances during and after exercise^(40)^. We hypothesised that athletes who perform sports requiring higher intensive exercise may have a shorter lifespan because of their high metabolic entropy generation. In the present study, we aim to elucidate the lifespan entropy generation of athletes based on the dietary recommendations for four different sports and compare the results of these calculations with the statistical lifespan data.

## Materials and methods

### Menu planning for the athletes

The energy needs of the athletes vary widely, depending on the activity. For instance, it is not uncommon for Tour de France cyclists to require more than 5000 kcal/d. The recent guideline suggests that women need approximately 1800–2200 kcal/d and men need approximately 2400–3000 kcal/d^(39)^. Energy expenditure is affected by the body composition and duration, intensity and frequency of exercise^(40)^. Approximately 2000 kcal/d of calories is needed by the general public, an additional 1100 kcal/d is needed by a runner and endurance athletes usually require more calories than weight lifters^(40)^. In the present study, diets were planned to provide 3000–3500 kcal/d for an exercise day (Table 2). We also assumed that when an athlete reaches a certain age, because of the lack of intense exercise he/she starts to eat as recommended for healthy individuals, i.e., 19–59 years old adults consume 1600–3000 kcal/d^(38)^. Data employed in this study were openly available for use and adapted from the references providing dietary recommendations by citing the source. We provided the restrictions, which apply to our calculations by making some assumptions. Lifespan is indeed a long time, and there will be inevitably some changes in the nutritional preferences of the athletes in this period. We believe that after the publication of this study, many other people will utilise the same method in their research; therefore, we wanted to provide as much detail to them as possible.

**Table 2.** Total calories and composition of the diet plans for different groups of athletes and a 50 years old healthy retired person.

	Cyclist	Weightlifter	Rugby player	Golfer	50 years old healthy retired person
Body weight of the athlete (kg)	67	90	105	70	
Total calorific uptake (kcal/kg day)	52⋅23	41⋅11	38⋅10	32⋅14	2000
Carbohydrates (g/kg day)	7⋅40	5⋅68	4⋅60	4⋅77	280
Proteins (g/kg day)	2⋅91	2⋅42	2⋅40	1⋅69	77
Fats (g/kg day)	1⋅22	1⋅03	1⋅17	0⋅71	66
Total carbohydrates, proteins and fats (g/kg)	11⋅5	9⋅13	8⋅17	7⋅17	423

Total calorific uptake, carbohydrates, proteins and fats supplied by the diet to the athletes are expressed per kg of the bodyweight of the athlete.

There is an ideal weight and height range for athletes to be successful in any sports. Wenzel Coaching^(41)^ indicated that the height range of the cyclist is 157–193 cm, the weight range of the male climbers is between 50 and 69 kg and those of the male sprinters is 60–90 kg. The average height and the weight of these athletes were 1⋅80 m and 68⋅8 kg, and in the present study, calculations are performed for an athlete with 67 kg body weight (Table 2). Wilk's score measures the strength in powerlifting against other powerlifters. A weightlifter with 90 kg of body weight with Wilk's score of 450 and 500 can compete in the national-level meet or at world-class, respectively^(42)^; calculations presented in Table 2 are valid for such a weightlifter. In the 2012/2013 season, the height and weight of the athletes of New Zealand's Blues rugby team were varying between 202–178 cm and 80–129 kg, respectively^(43)^. In Table 2, assessments have been done for an average-weight rugby player.

People with varying heights and weights may be professional golfers. Irish golfer Rory McIlroy was a good PGA Tour golfer in 2010 and rose to No. 1 in the world by 2012, he was 178 cm tall and 73 kg. Tiger Woods was 188 cm tall and 70 kg when he turned pro in 1996.

He is widely regarded as one of the greatest golfers of all time and one of the most famous athletes in history and elected to the World Golf Hall of Fame in 2020. Gary Woodland was voted the most athletic golfer on the PGA tour in 2011. He was 185 cm tall and 91 kg^(44)^. The golfer, for whom the calculations are performed in Table 2, had exactly the same bodyweight as Tiger Woods and similar body weight as Rory McIlroy.

### Thermodynamic considerations

**Fig. 1.**
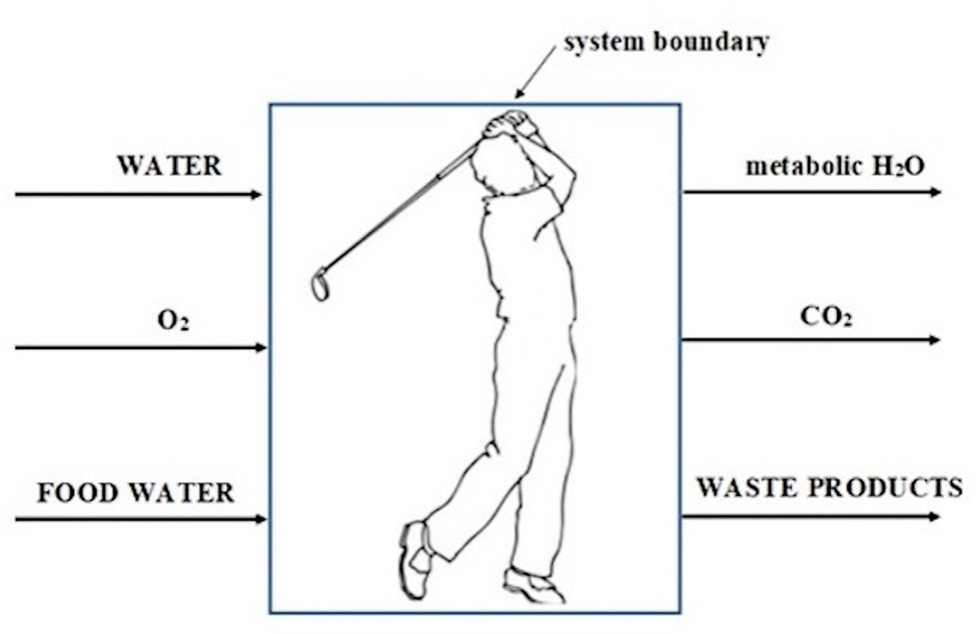
Description of the human body as a thermodynamically open system.

Fig. 1 illustrates the athlete's body as a thermodynamically open system. While calculating the entropy generation by the athletes the same procedure is employed as Öngel *et al.*^(20)^, a healthy person was assumed to digest 99 % of the carbohydrates, 95 % of the lipids and 92 % of the proteins. We calculated entropy generation during the metabolism of carbohydrates, lipids and proteins in terms of glucose, palmitic acid and the average of 20 amino acids based on the following equations:
1
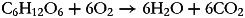

2
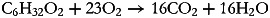

3
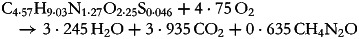

Describing the metabolism with equations (1–3) is an oversimplification, during exercise lactic acid accumulates in the muscles of the athletes and then it is reused. Energy, e.g., enthalpy, extracted from carbohydrates will not be affected by lactic acid accumulation and its reuse in the muscles, since enthalpy is a state function and its value depends on the initial, carbohydrates and the oxygen, and the final chemical species, e.g., carbon dioxide and water^(45)^. Ulu *et al.*^(46)^, while discussing energy storage and reuse in biological systems, reported that entropy generation associated with this phenomenon is extremely small and does not contribute to the lifespan entropy accumulation; therefore, entropy generation during lactic acid utilisation and reuse in the muscles is neglected.

Metabolic waste after digestion and absorption processes was removed with the urine and the faeces. Following the same procedure as Öngel *et al.*^(20)^, the amount of urine was determined to limit its urea content as 20 g/l and 65 % of urea is excreted. The amount of faeces excretion was calculated by equation (4). It was assumed that the athletes consume 3⋅0 litres/d water and non-athlete healthy people drink 2⋅0 litre water daily.
4
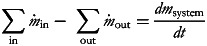

where *dm*_system_/*dt*, i.e., water retention in the body, was assumed zero.

The amounts of the inhaled O_2_, excreted CO_2_, H_2_O and the wastes excreted from the body for each athlete are shown in Table 3.

**Table 3.** The amounts of the inhaled O_2_, excreted CO_2_ and H_2_O, urine and faces and metabolic heat production, work performance and the total heat loss from the body during the active years and after retirement

	Cyclist	Weightlifter	Rugby player	Golfer	After retirement
O_2_ (g/d)	977	1050	1142	628	566
H_2_O (g/d)	470	501	532	305	272
CO_2_ (g/d)	1195	1277	1360	774	682
Faeces (g/d)	1228	1019	709	1972	1303
Urine (g/d)	1856	2075	15 885	1080	733
Metabolic heat production (kJ/d)	13 549	14 584	9428	8686	7691
Work performance (kJ/d)	3709	3950	3716	2413	2247
Heat loss (kJ/d)	11 126	11 850	11 147	7238	6740

In the present study, calorie uptake with each food is calculated in terms of their enthalpies exactly the same way as Öngel *et al.*^(20)^ In biological systems, the molecular structures are usually so sophisticated that their thermodynamic properties are not readily available in the tables and usually calculated with the group contribution method^(47)^. The best-known group contribution methods were suggested by Joback and Reid^(48)^. According to this method, a complex biological structure is separated into its contributing chemical sub-groups, then thermodynamic properties of each sub-group are evaluated individually. The thermodynamic property of the complex chemical structure is calculated by summing up the thermodynamic properties of its sub-groups. For instance, sucrose is a disaccharide, composed of two monosaccharides glucose and fructose. The standard enthalpies of the formation of glucose and fructose are −1271 and −1265 kJ/mol; when we add up these numbers, we estimate the enthalpy of the formation of sucrose according to the group contribution method as −2536 kJ/mol. In the standard thermodynamic tables, the enthalpy of formation of sucrose is given as −2226 kJ/mol. The difference between the estimate and the experimentally determined value is approximately 12 %. While carrying out such calculations, only metabolisable chemicals are employed. Any non-digestible chemical, which is available in the food is not included in the calculations. Sucrose, glucose and fructose have relatively simple structures, and their thermodynamic properties are available in the tables; on the other hand, with the extremely sophisticated structures, referring to the group contribution method is the only way to proceed^(47)^.

By assuming that the nutrients intake into the body is at 25°C, and H_2_O and CO_2_ exit at 37°C, energy balance was calculated via equation (5). The energy balance around the human is:
5
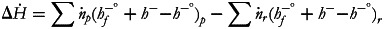

where 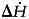 is the enthalpy release via equation (5), 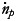 and 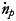 represent the mole number rates of the products output from and the reactants input to the system, respectively; parameters 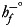, *h*^−^ and 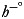 are the formation enthalpies at the standard conditions. The thermodynamic properties of the compounds are shown in Table 4. It was assumed that 32, 106 and 8 moles of ATP were created by the metabolism of one mole of each of glucose, palmitic acid and the average 20 amino acids, respectively. Total work achieved by the formation of the ATP molecules was found with equation (6); 34⋅6, 32⋅2 and 10⋅4 % represent the metabolic efficiency (*η*) of glucose, palmitic acid and the average of 20 amino acids, respectively^(17,18)^.
6
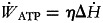

where 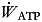 represents the total work performance rate via ATP utilisation.

**Table 4.** Thermodynamic properties of the nutrients and the products of the metabolism at 1 atm (adapted from Kuddusi)^(19)^

Chemical	Enthalpies and entropies of the nutrients, O_2_, CO_2_ and H_2_O at 1 atm			
	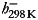 (kJ/kmol)	s at (298 K) (kJ/kmol K)	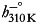 (kJ/kmol)	s at (310 K) (kJ/kmol K)
C_6_H_12_O_6_ (glucose)	−1260 × 10^3^	212	–	
C_16_H_32_O_2_ (palmitic acid)	−835 × 10^3^	452	–	
C_4⋅57_H_9⋅03_N_1⋅27_O_2⋅25_S_0.046_ (average of the 20 amino acids)	−385 × 10^3^	1.401 × 119	–	
O_2_	8682	218		220
H_2_O			10 302	219
CO_2,_			9807	243

Total heat loss from the skin and through respiration was calculated from equations (7) and (8) with the conversion rate recommended by Hall^(49)^ and presented in Table 3.
7
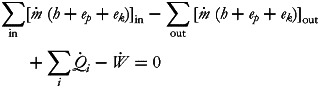

8
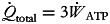


### Entropy balance

In accordance with the descriptions in the literature, the weights of a male cyclist, weightlifter, rugby player and golfers were assumed 67, 85, 105 and 70 kg, respectively. The entropy generation rate of the athletes was calculated depending on their diets by equation (9) and is given in Table 5.
9
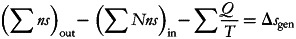

where *n* describes the mole number of chemicals taken in or excreted from the system, *s* is the entropy of the chemicals, *T* is the human body temperature as 37°C and *Q* is the heat generated by the body. Daily consumption of the amounts of carbohydrates, lipids and proteins uptake are shown in Table 2 made it possible to calculate the amounts of the O_2_ breathed in O_2_, respired and CO_2_ and H_2_O exhaled and the excreted waste (Table 3).

**Table 5.** The total entropy generation rate and the expected lifespan of the athletes

	Cyclist	Weightlifter	Rugby player	Golfer
Daily entropy generation rate (kW/kg K) in case of consumption of the athletes’ diet	6⋅4 × 10^−6^	5⋅5 × 10^−6^	4⋅8 × 10^−6^	3⋅9 × 10^−6^
Annual entropy generation rate (kJ/kg K) in case of consumption of the athletes’ diet	200	170	150	122
Total entropy generation (kJ/kg K) until retirement	10 010	8491	7514	6121
Lifespan (years) in case of consumption of the athletes’ diet throughout the lifespan	56	66	75	92
Daily entropy generation rate (kW/kg K) in case of consumption of the retirement diet	3⋅5 × 10^−6^	2⋅8 × 10^−6^	2⋅3 × 10^−6^	3⋅4 × 10^−6^
Annual entropy generation rate (kJ/kg K) in case of consumption of the retirement diet after retiring	110	87	70	106
Lifespan (years) in case of consuming the athletes’ diet for 50 years and then consuming the retirement diet	63	84	105	100

The athletes were assumed to retire when they become 50 years old and start consuming a special diet prepared for them. The lifespan entropy generation limit was assumed to be 11 404 kJ/K kg^(6,17)^, and the athletes die when they come to this limit. Diet plans have been designed in the present study by referring to the nutritional guidelines and scientific articles to provide sufficient nutrients for each athlete, and then the lifespan entropy has been calculated as presented in Table 5. These calculated lifespans are then compared to the observed longevities to be sure that the thermodynamic model is correct.

## Results and discussions

In the present study, we prepared four different diets for cyclists, weightlifters, rugby players and golfers to consume during their active sports careers and another diet was written for the retirement period of their life. Cycling is among the high-static and high-dynamic component sports (Table 1). Therefore, oxygen uptake and metabolic activity of the cyclists are higher than those of the athletes involved in other sports such as weightlifting, rugby and golf. The results of the present study indicate that the oxygen uptake of a cyclist would be 977 g/d and his metabolic heat generation would be 13 549 kJ/d. The results of this study suggest that a cyclist may live 56 years and generate 6⋅4 × 10^−6^ kW/kg K per day and 200 kJ/kg K per year of entropy (Table 5) when he consumes the special diet for cyclists’ diet. If he should change his diet after retirement, he may live 6⋅4 years longer and generate 2⋅9 × 10^−6^ kW/kg K of daily and 89⋅8 kJ/kg K less annual entropy (Table 5). A cyclist produces the highest entropy in comparison with a weightlifter, rugby player and golfer. This metabolic difference results from the weight difference between the athletes and different amounts of calorie intake and diet composition. Martin *et al.*^(50)^ advise cycling as physical activity for people due to their health benefits. Likewise, Sanchis-Gomar *et al.*^(51)^ found that 50 % of the general population died at 73⋅5 when Tour de France participants had an average lifespan of 81⋅5 years. According to our findings, the lifespan of cyclists doing high-intensity exercise was lower than others because of the high entropy generation rate. However, these results should be evaluated with caution since the athletes’ body composition, muscular development, mental health and physiological changes were not taken into consideration in the present analysis.

Weightlifting has high-static (>50 % maximal voluntary contraction (MVC)) and low-dynamic (<40 % Max O_2_) components as shown in Table 1. The present results show that a weightlifter consumes 1050 kg/d of O_2_ and generates the highest amount of metabolic heat with 14 584 kJ/d during his active sports life as an athlete (Table 3). He generates 5⋅5 × 10^−6^ kW/kg K per day and annual 169⋅8 kJ/kg K per year entropy; therefore, if he remains on the weightlifter diet until the end of his lifespan, he may live until 66 years of age. However, if he consumes the retirement diet for the rest of his life, his remaining life expectancy may increase by 17⋅40 years (Table 5). Huebner *et al.*^(52)^ argue that Olympic weightlifting results in high power output; therefore, explosive power, speed and strength are necessary for it and the prevalence of metabolic disorders is low in weightlifters due to the effects of physical activity on human health. In this study, it is found that the work performance of the weightlifter is the highest with 3950 kJ/kg per day among the four groups we studied.

Rugby sport requires 20–50 % MVC and 40–70 % of the Max O_2_ (Table 1). A rugby player inhales 1142 g/d of O_2_ during his active sports life (Table 3). The present results indicated that, on average, a rugby player may live 105 years, this is longer than the expected average lifespan of the cyclists, weightlifters and golfers, if he consumes the athletes’ diet prepared for him until the age of 50, and after this age the retirement diet (Table 5). If he continues to consume the athletes’ diet, his life expectancy may decrease for 30 years and on average he may generate 4⋅8 × 10^−6^ kW/kg K of daily and 150 kJ/kg K of annual entropy. If the rugby player should choose to uptake the same calorie and have the same composition diet in his retirement, he may generate lower daily nutritional entropy per weight, i.e., 2⋅3 × 10^−6^ kW/kg K. This is lower than the daily entropy generation rates of the cyclist, weightlifter and the golfer. Davies *et al.*^(53)^ found that the prevalence of diabetes and cardiovascular disease and hypertension was lower in elite former rugby players at the age of 50+, and the anxiety and musculoskeletal disorder were higher than the English Longitudinal Study of Aging participants. This may be interpreted like that the rugby players generally do not suffer from metabolic diseases and their longevity is not reduced due to anxiety or musculoskeletal morbidity.

As shown in Table 1, the percent of Max O_2_ and MVC of golf is lower than that of a cyclist, weightlifter or rugby player. According to the present results, golfers consume a lower amount of oxygen, their metabolic heat generation, work performance and heat loss via respiration and perspiration during their active life period is lower in comparison with cyclists, weightlifters and rugby players (Table 3). The present results also showed that if the athletes consume the special diets for their active life periods during their whole life, golfers’ daily entropy generation rate, i.e., 3⋅9 × 10^−6^ kW/kg K, would be lower than those of the other athletes and thus, their lifespan expectancy, 91⋅9 years, would be higher than those of the others (Table 5). If they should consume the retirement diet in their retirement years, a golfer may live for 100 years and generate 3⋅4 × 10^−6^ kW/kg K entropy daily (Table 5). Although our assessment was based on the assumption that the athletes changed their diet at the age of 50, upon retirement, if they should finish their active career at an earlier age, they may shift to a retirement diet earlier, and under these circumstances, their longevity may increase more than what is reported in Table 5. According to Farahmand *et al.*^(54)^, golfers have the lowest mortality rate, due to the lower intensity of exercise in golfing sport, when compared with the exercise performed by other athletes. Murray *et al*.^(55)^ suggested that golf positively contributes to life expectancy, because it is a medium intensity aerobic physical activity, playing golf is beneficial to the health of the elder players because it involves walking exercise.

Hayflick established the entropic age concept^(7–10)^, and Annamalai and co-workers improved it significantly^(6,17,18)^ by working on numerical examples and estimating the ageing stress on each organ^(18)^. Kuddusi^(19)^, Öngel *et al.*^(20)^ and Patel and Rajput^(56)^ made a further contribution to the entropic age concept via applying it to different diets. Every athlete consumes a specially designed diet to be successful. Different longevities are given in the literature for the athletes participating in different sports. In the present study, a perfect agreement was found between the observed and the athletes’ special diet-based longevity estimates.

The present results are based on the nutritional entropy generation and do not show the exact lifespan of athletes such as rugby players, cyclists, weightlifters and golfers, because there are also several other factors such as disease, mental condition, bodily injury and genetic factors affecting the athletes’ life expectancy. In a recent study, Öngel *et al.*^(57)^ reported how a disease may affect the entropy generation in a patient's lifespan.

In his Nobel-winning study, Schrödinger^(58)^ explains that living systems import energy and export entropy to maintain their lives. Internal and external work performance and entropy generation by the living systems are studied in detail by Semerciöz *et al.*^(59)^. Yildiz *et al.*^(60)^ elaborated the entropy generation and entropy accumulation concept by the living system further. The work performance, entropy generation and accumulation concepts as discussed by these authors also apply to this study. Semerciöz *et al.*^(59)^ refer to the work performed by the lungs, heart, liver, kidneys, nervous system and manufacturing of the bones, and muscles are ‘internal work’. If the athlete is pregnant, work performed by her to synthesise the baby is also regarded as internal work. All the work, leading to consequences observable with eye from the outside of the system, such as chewing, running and walking, are ‘external work’. An athlete may perform all of these kinds of work by utilising ATP.

## Conclusion

It was reported in the literature that the life expectancy of the athletes may be affected by the static and dynamic components of the sports they perform.^(61)^ After employing the diet-based entropic assessment method the same way as recently reported by Öngel *et al.*^(20)^, we suggest that calorie and composition of the diet affect the metabolic entropy generation rates of the athletes depending on their metabolism. This study may be a starting point of the forthcoming medical studies on the athletes’ health, longevity and morbidity. The expected average longevity for male athletes was 56 years for a cyclist, 66 years for a weightlifter, 75 years for a rugby player and 92 years for a golfer. If they should start consuming the retirement diet after 50 years of age, the longevity of the cyclist may increase for 7 years, and those of weightlifter, rugby player and golfer may increase for 22, 30 and 8 years, respectively.
